# Rosamines Targeting the Cancer Oxidative Phosphorylation Pathway

**DOI:** 10.1371/journal.pone.0082934

**Published:** 2014-03-12

**Authors:** Siang Hui Lim, Liangxing Wu, Lik Voon Kiew, Lip Yong Chung, Kevin Burgess, Hong Boon Lee

**Affiliations:** 1 Drug Discovery Laboratory, Cancer Research Initiatives Foundation (CARIF), Subang Jaya, Selangor, Malaysia; 2 Department of Pharmacy, University of Malaya, Kuala Lumpur, Malaysia; 3 Department of Chemistry, Texas A & M University, College Station, Texas, United States of America; 4 Department of Pharmacology, Faculty of Medicine, University of Malaya, Kuala Lumpur, Malaysia; University of Windsor, Canada

## Abstract

Reprogramming of energy metabolism is pivotal to cancer, so mitochondria are potential targets for anticancer therapy. A prior study has demonstrated the anti-proliferative activity of a new class of mitochondria-targeting rosamines. This present study describes *in vitro* cytotoxicity of second-generation rosamine analogs, their mode of action, and their *in vivo* efficacies in a tumor allografted mouse model. Here, we showed that these compounds exhibited potent cytotoxicity (average IC_50_<0.5 µM), inhibited Complex II and ATP synthase activities of the mitochondrial oxidative phosphorylation pathway and induced loss of mitochondrial transmembrane potential. A NCI-60 cell lines screen further indicated that rosamine analogs **4** and **5** exhibited potent antiproliferative effects with Log_10_GI_50_ = −7 (GI_50_ = 0.1 µM) and were more effective against a colorectal cancer sub-panel than other cell lines. Preliminary *in vivo* studies on 4T1 murine breast cancer-bearing female BALB/c mice indicated that treatment with analog **5** in a single dosing of 5 mg/kg or a schedule dosing of 3 mg/kg once every 2 days for 6 times (q2d×6) exhibited only minimal induction of tumor growth delay. Our results suggest that rosamine analogs may be further developed as mitochondrial targeting agents. Without a doubt proper strategies need to be devised to enhance tumor uptake of rosamines, i.e. by integration to carrier molecules for better therapeutic outcome.

## Introduction

Conventional cancer chemotherapy depends on drugs that act by interrupting DNA replication, i.e. by inhibiting the synthesis or function of new nucleic materials, or by causing irreparable damage to vital nucleic acids through intercalation, alkylation or enzymatic inhibition. Lack of selectivity for neoplastic cells exhibited by these drugs typically limits their success as treatment agents. Contemporary chemotherapeutic strategies that target signaling pathways or particular gene products tend to be limited to cancers driven by a dominant oncogene and are often vulnerable to resistance via the multiplicity of tumorigenesis signaling pathways [1]. For example, most of the HER-2 positive metastatic breast cancer patients who initially responded to treatment with trastuzumab develop secondary trastuzumab resistance within a year after the treatment began [2]. Similar observations have been made for BRAF-targeted vemurafenib for melanoma therapy [3] and EGFR-targeted gefitinib or erlotinib for the treatment of non-small cell lung cancer [4].

A relatively new alternative to targeting DNA and enzymes in rapidly proliferating cells, or specific signaling pathways is to focus on organelles like the mitochondria. Mitochondria are the energy generators that maintain cell life and essential cell functions, including multiple signaling cascades that regulate cells, for instance, metabolism, cell cycle control, development, and cell death [5]. In cancer chemotherapy, mitochondria-targeting drugs interfere with cancer cell metabolisms by pertubing the mitochondrial transmembrane potential, inhibiting the electron redox chain complexes, interfering the mitochondria transmembrane permeability, and targeting mitochondrial-DNA [6], . The most common types of mitochondria-targeting drugs are lipophilic, cationic drugs; these are selective for cancer cells because they tend to have higher mitochondrial membrane potentials than normal epithelial cells [8], [9].

**Figure 1:**
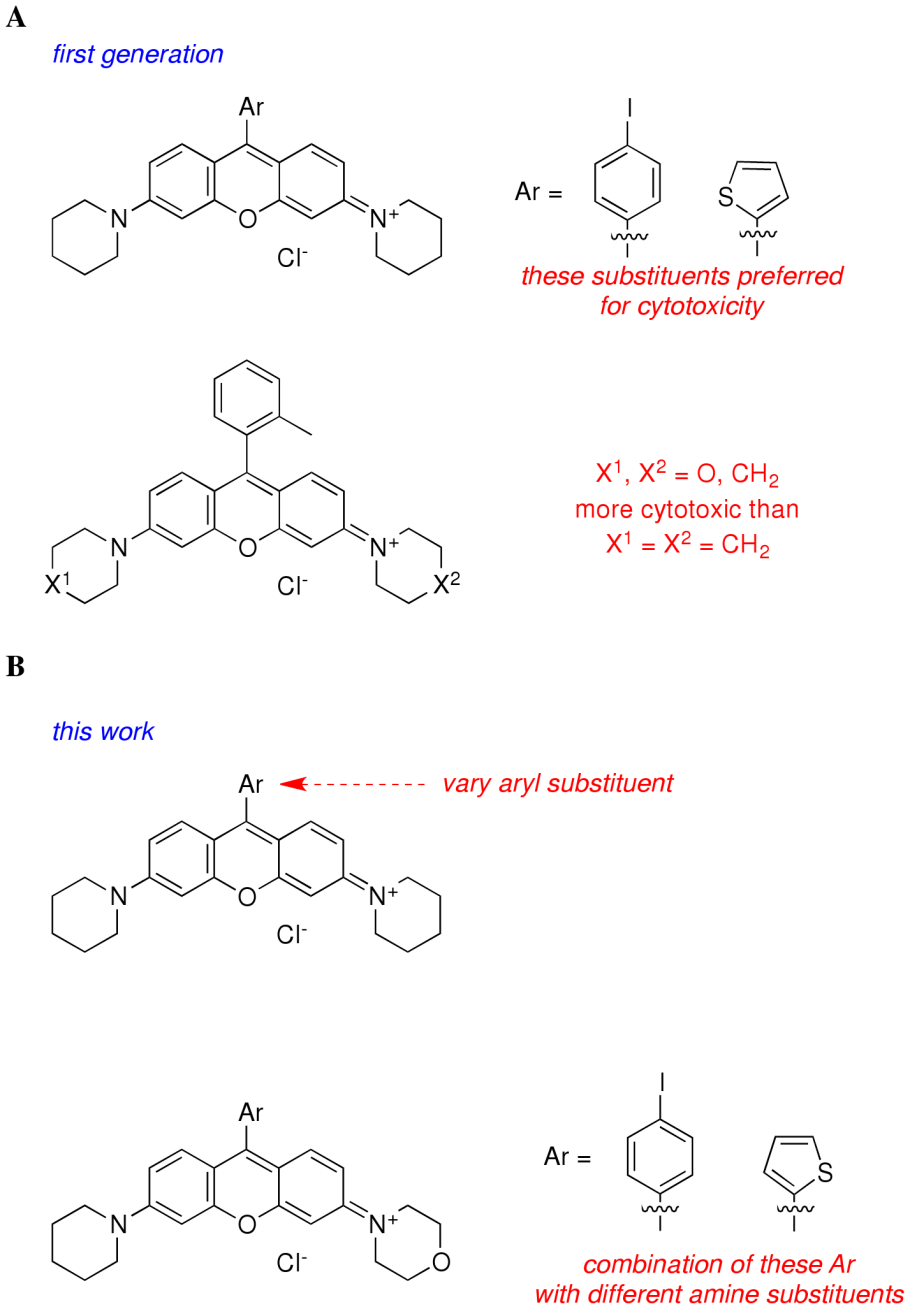
Structural variations of rosamine. (A) of the rosamine dyes variously functionalized at the *meso* position as previously reported by Lim et al. (Anticancer Drugs 2009, 20: 461–468), the ones shown here with *meso*- thiofuran or 4-iodophenyl had superior anticancer activities in cellular assays. (B) Second-generation targets featured in this work.

We have previously reported structure-activity relationships (SARs) for the anticancer properties of a series of rosamines (Fig. 1A) [10], [11]. These compounds are delocalized lipophilic cations (DLC) with peripheral cyclic amines and various groups at the *meso* position; the majority of these cations are substantially more potent than rhodamine-123 which is a well-studied delocalized lipophilic cation. Consistence with this, SAR data for the rosamines suggested good potencies correlated with hydrophobic cyclic amines and *meso*-aryl substituents. Thiofuryl and 4-iodophenyl *meso* substitution corresponded to approximately 5-fold improvement in potency compared to phenyl substitution. Additionally, the data indicated that significantly lower IC_50_ values were obtained for unsymmetrical compounds (X^1^ not same as X^2^), but the combination of unsymmetrical amine substituents with thiofuryl and 4-iodophenyl *meso* substitution was not explored. Intracellular imaging on representative examples indicated these compounds accumulate in the mitochondria. These compounds (i.e rosamine **2** and **5**) are also found to be more cytotoxic against cancer cells compared with immortalized normal epithelial cells of the same organ type [10].

The research reported here was undertaken to probe how two molecular modifications affect cytotoxicities in this series: (i) replacement of *para*-iodo or thiofuran groups with other *para*-halide or furan groups; and, (ii) combination of thiofuryl and 4-iodophenyl *meso* substitution with piperidine/morpholine combinations. Thus we report syntheses of second generation rosamines (Fig. 1B) and their cytotoxicities relative to a panel of solid tumor cell lines. Compounds with promising activity were further evaluated in the NCI-60 human tumor cell lines screen. Selected rosamines were also examined for their effect on cellular redox systems and for effects in an *in vivo* tumor model.

## Materials and Methods

### Ethics Statement

All animal experiments were conducted in accordance with protocols reviewed and approved by Dr. Haji Azizuddin Bin Haji Kamaruddin, Laboratory Animal Centre (LAC) Animal Care and Use Committee of Faculty of Medicine, University of Malaya (reference number FAR/14/07/2010/LSH). Female BALB/c mice aged between 6–8 weeks with a minimum weight of 17 g were maintained in a controlled environment of 12 h light-dark cycles with free access to food (standard pellet diet purchased from Altromin International, Lage, Germany) and purified water. Female mice were used because the hormonal environment essential for development of implanted 4TI mouse mammary carcinoma would be present.

### Materials

Minimum essential medium with Earl's salt and L-glutamine, RPMI 1640 medium with L-glutamine, fetal bovine serum, Pen-Strep (10 000 U/ml penicillin, 10 mg/ml streptomycin), trypsin 10× were supplied by GIBCO, Invitrogen (Auckland, New Zealand). JC-1 was purchased from Molecular Probes, Invitrogen (Oregon, USA). Dimethyl sulfoxide (DMSO), hydrochloric acid 37%, polyethylene glycol 400 (PEG 400) and 2-propanol were purchased from Merck (Hohenbrunn, Germany). Ethylene glycol*bis*(aminoethylether)-*tetra*-acetic acid (EGTA), ethylenediaminetetraacetic acid (EDTA), 3-(*N*-morpholino)propanesulfonic acid (MOPS), potassium chloride, potassium dihydrogen orthophosphate, sodium chloride, sodium hydrogen carbonate, disodium hydrogen orthophosphate anhydrous, sucrose and Tris were purchased Fisher Scientific (Leicestershire, UK). Thiazoyl blue tetrazolium bromide (MTT) was purchased from Amresco (Ohio, USA). Saline (0.9% sodium chloride) was obtained from Duopharma Sdn. Bhd. (Selangor, Malaysia). Carbonyl cyanide 3-chlorophenylhydrazone (CCCP) was purchased from Sigma (Steinheim, Germany). Protein assay dye reagent concentrate was obtained from Bio-Rad Laboratories (California, USA). Complex I, Complex II, Complex IV and ATP synthase enzyme activity microplate assay kit were purchased from MitoSciences (Oregon, USA).

### Syntheses of Rosamine Analogs

**Figure 2:**
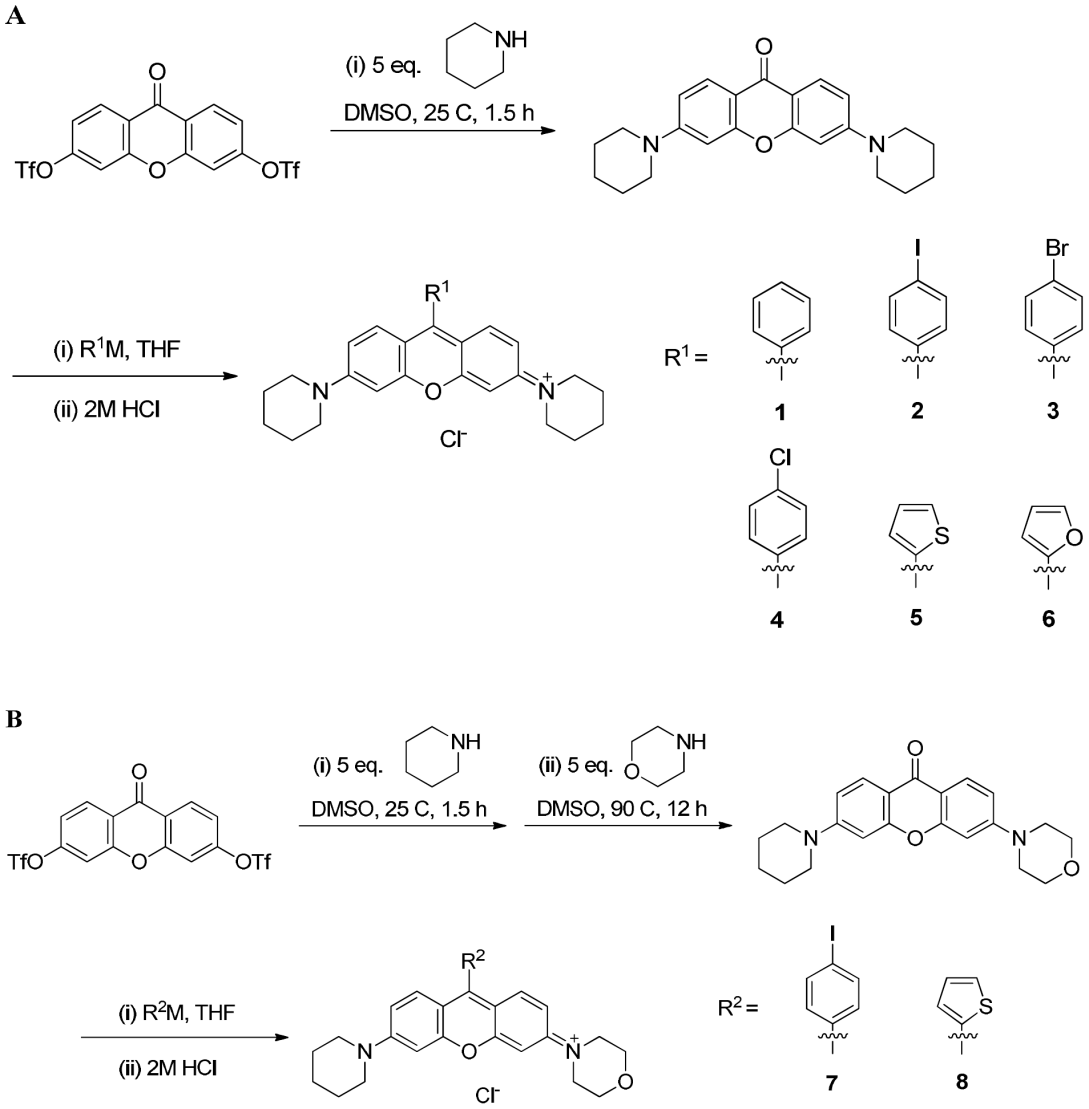
Schematic illustration of the synthesis of rosamines. (A) The starting material of xanthone ditriflate was prepared in solution by triflation of the phenols, followed by animation of the triflate with piperidine to give symmetrical cyclic amines substitution or; (B) by stepwise addition of piperidine and morpholine to give unsymmetrical cyclic amines substitution.

Rosamines were synthesized and purified as described previously [11]. The starting material of xanthone ditriflate was prepared in solution by triflation of the phenols, followed by animation of the triflate with piperidine to give symmetrical cyclic amines substitution (Fig. 2A) or by stepwise addition of piperidine and morpholine to give unsymmetrical cyclic amines substitution (Fig. 2B) [11]. The resulted 3,6-diamino-xanthen-9-one was reacted with organolithium or Grignard reagents to yield the desired rosamine structures.

In general, a Grignard reagent or lithium reagent (1.0 mmol) was added dropwise over 1 min to the solution of 0.2 mmol 3,6-diamino-xanthen-9-one in 5 ml of THF at 0°C. The reaction mixture was stirred for 12 h at room temperature. After completion of reaction, 2 ml of 2 M aqueous HCl was added, stirred for 10 min to quench the reaction and diluted with 20 ml of CH_2_Cl_2_. The organic layer was washed with water and brine, dried over anhydrous Na_2_SO_4_, and concentrated under reduced pressure. The residue was purified by flash chromatography (5% to 10% MeOH/CH_2_Cl_2_) to give the pure product.

Spectral data for the synthesized compounds are listed here except the synthesis and spectral data for rosamines **1**, **2** and **5** were as previously reported [11]. They are named below in a way that describes the *meso-*substituents and assumes the compound is symmetrical unless otherwise indicated.

#### 4-Bromobenzene Rosamine 3

^1^H NMR (300 MHz, CDCl_3_) δ 7.72 (d, 2H, *J* = 8.4 Hz), 7.27–7.21 (m, 4H), 7.09 (dd, 2H, *J* = 9.6, 2.6 Hz), 6.94 (d, 2H, *J* = 2.6 Hz), 3.74–3.70 (m, 8H), 1.74 (br, 12H); ^13^C NMR (75 MHz, CDCl_3_) δ 158.1, 156.3, 155.0, 132.2, 131.6, 130.9, 130.5, 124.9, 115.0, 113.3, 97.4, 49.1, 25.9, 24.0; λ_max abs_ 570 nm, λ_max emiss_ 590 nm, ε 110800 M^−1^ cm^−1^, fwhm 38 nm, Φ 0.78±0.02 in CH_2_Cl_2_; HRMS (ESI) m/z calcd for (M-Cl)^+^ C_29_H_30_BrN_2_O 501.1542; found 501.1539.

#### 4-Chlorobenzene Rosamine 4

^1^H NMR (300 MHz, CDCl_3_) δ 7.56 (d, 2H, *J* = 8.4 Hz), 7.30 (d, 2H, *J* = 8.4 Hz), 7.26 (d, 2H, *J* = 9.6 Hz), 7.09 (dd, 2H, *J* = 9.6, 2.4 Hz), 6.95 (d, 2H, *J* = 2.4 Hz), 3.74–3.70 (m, 8H), 1.74 (br, 12H); ^13^C NMR (125 MHz, CDCl_3_) δ 158.1, 156.3, 155.1, 136.7, 131.6, 130.8, 130.1, 129.3, 115.0, 113.4, 97.5, 49.2, 25.9, 24.1; λ_max abs_ 570 nm, λ_max emiss_ 590 nm, ε 119100 M^−1^ cm^−1^, fwhm 38 nm, Φ 0.80±0.02 in CH_2_Cl_2_; HRMS (ESI) m/z calcd for (M-Cl)^+^ C_29_H_30_ClN_2_O 457.2047; found 457.2052.

#### Furan Rosamine 6

^1^H NMR (500 MHz, CDCl_3_) δ 7.97 (d, 2H, *J* = 9.7 Hz), 7.92–7.91 (m, 1H), 7.18 (dd, 2H, *J* = 9.7, 2.6 Hz), 7.15–7.14 (m, 1H), 6.90 (d, 2H, *J* = 2.6 Hz), 6.82–6.81 (m, 1H), 3.74–3.72 (m, 8H), 1.76 (br, 12H); ^13^C NMR (125 MHz, CDCl_3_) δ 158.2, 156.0, 147.3, 145.7, 142.2, 132.0, 120.5, 115.0, 113.3, 111.8, 97.5, 49.0, 25.9, 24.1; λ_max abs_ 592 nm, λ_max emiss_ 627 nm, ε 86900 M^−1^ cm^−1^, fwhm 105 nm, Φ 0.38±0.01 in CH_2_Cl_2_; HRMS (ESI) m/z calcd for (M-Cl)^+^ C_27_H_29_N_2_O_2_ 413.2229; found 413.2232.

#### Unsymmetrical 4-Iodophenyl Rosamine 7

^1^H NMR (300 MHz, CDCl_3_) δ 7.96 (d, 2H, *J* = 8.3 Hz), 7.35–7.29 (m, 2H), 7.17–7.09 (m, 6H), 3.88 (t, 4H, *J* = 4.7 Hz), 3.78–3.71 (m, 8H), 1.77 (br, 6H); ^13^C NMR (75 MHz, CDCl_3_) δ 158.6, 157.9, 156.9, 156.7, 155.9, 138.2, 131.9, 131.5, 131.1, 131.0, 115.5, 114.6, 114.0, 113.7, 98.6, 97.9, 97.1, 66.4, 49.5, 47.4, 26.1, 24.0; λ_max abs_ 565 nm, λ_max emiss_ 586 nm, ε 80900 M^−1^ cm^−1^, fwhm 39 nm, Φ 0.52±0.02 in CH_2_Cl_2_; HRMS (ESI) m/z calcd for (M-Cl)^+^ C_28_H_28_IN_2_O_2_ 551.1195; found 551.1192.

#### Unsymmetrical Thiofuryl Rosamine 8

^1^H NMR (500 MHz, CDCl_3_) δ 7.69 (dd, 1H, *J* = 4.8, 1.4 Hz), 7.62 (d, 1H, *J* = 9.7 Hz), 7.59 (d, 1H, *J* = 9.7 Hz), 7.26–7.22 (m, 2H), 7.19 (dd, 1H, *J* = 9.7, 2.5 Hz), 7.09 (dd, 1H, *J* = 9.7, 2.5 Hz), 7.00 (d, 1H, *J* = 2.5 Hz), 6.90 (d, 1H, *J* = 2.5 Hz), 3.78 (t, 4H, *J* = 4.9 Hz), 3.68–3.65 (m, 8H), 1.67 (br, 6H); ^13^C NMR (75 MHz, CDCl_3_) δ 158.1, 157.5, 156.6, 156.3, 149.8, 132.2, 132.0, 131.7, 130.6, 130.6 (two peaks: 130.62, 130.56), 128.2, 115.2, 114.6, 114.3, 114.0, 97.8, 97.2, 66.2, 49.1, 47.2, 25.9, 23.9; λ_max abs_ 576 nm, λ_max emiss_ 599 nm, ε 87600 M^−1^ cm^−1^, fwhm 42 nm, Φ 0.30±0.01 in CH_2_Cl_2_; HRMS (ESI) m/z calcd for (M-Cl)^+^ C_26_H_27_N_2_O_2_S 431.1793; found 431.1795.

### Cell Culture and *In Vitro* Cell Viability Assay

MCF-7 breast carcinoma and HCT-116 colon carcinoma cell lines were obtained from American Tissue Culture Collection (Virginia, USA) and maintained in RPMI 1640 medium supplemented with 10% FBS. HSC-2 oral cavity human squamous carcinoma cells were obtained from Health Science Research Resources Bank (Japan). HK-1, a previously characterized nasopharyngeal squamous carcinoma cells [12] is a gift by Prof. Wong Y.C. from the University of Hong Kong. Both cell lines were grown in MEM medium supplemented with 10% FBS. For cell viability assay, cells at 4000 cells/well in 80 µl of medium were inoculated in 96-well plates and were allowed to adhere overnight. Cells were then treated with each compound at concentrations ranging from 0.001–10 µM giving the final volume of 100 µl in each well. At the end of incubation period, 15 µl of 5.0 mg/ml MTT in phosphate buffered saline (PBS) was added and incubated for 4 h. Medium and excessive MTT were aspirated and the resulting formazan was solubilized with 100 µl of dimethyl sulfoxide. Absorbance was read at 570 nm with SpectraMax M4 microplate spectrophotometer (Molecular Devices, CA).

### NCI-60 Human Tumor Cell Line Screen

NCI-60 cell panel screening was performed by the NCI/National Institutes of Health developmental therapeutics program (Bethesda, US). This platform allows the determination of growth inhibitory profiles of test compounds on 60 different human cancer cell lines, representing leukemia, melanoma and cancers of the lung, colon, central nervous system, ovary, breast, prostate, and renal subpanel. Sulforhodamine B assay was used to assess the cytotoxicity of test agents in a panel of 60 cell lines [13]. Briefly, the human tumor cell lines of the cancer screening panel were grown in RPMI 1640 medium containing 5% FBS and 2 mM L-glutamine. For a typical screening experiment, cells were inoculated into 96 well microtiter plates in 100 µl at plating densities depending on the doubling time of individual cell lines. After cell inoculation, the microtiter plates are incubated at 37°C, 5% CO_2_ and 100% relative humidity for 24 h. Following incubation, aliquots of 100 µl of compound at different dilutions were added to the appropriate microtiter wells and were further incubated for 48 h. At the assay end-point, cells were fixed with trichloroacetic acid followed by Sulforhodamine B staining for cellular protein content. Sulforhodamine B absorbance was read at a wavelength of 515 nm as a measurement of cell density.

### Mitochondria Isolation and Detergent Solubilization

Functional mitochondria were isolated from mouse liver by differential centrifugation method [14]. Briefly, a mouse (∼30 g) was starved overnight before sacrificed by cervical dislocation. The liver was harvested promptly and rinsed with ice-cold mitochondria isolation buffer (10 mM Tris-MOPS, 1 mM EGTA/Tris, 0.2 M sucrose, pH 7.4) until blood-free. The liver was then cut into small pieces in a beaker using scissors while keeping in an ice-bath. The buffer was replaced with 5 ml of fresh isolation buffer and the liver was homogenized with a Polytron probe (Ultra-Turrax T8, Ika-Werke, Germany) until smooth. The homogenate was centrifuged at 1000 g for 15 min at 4°C. The pellet was discarded and the supernatant was centrifuged at 12000 g for 15 min at 4°C to pellet the mitochondria. The mitochondria were washed twice by resuspending in 4 volumes of isolation buffer containing 1× protease cocktail inhibitor. The concentration of the mitochondrial protein was determined using the Bradford (Biorad protein assay) method. The mitochondria were frozen in 10 mg/ml aliquot at −80°C.

Detergent solubilization of the mitochondria proteins was done prior to measurement of the oxidative phosphorylation complexes activity. Mitochondria were diluted to 5.5 mg/ml with PBS and solubilized by addition of a 1/10th volume of detergent provided to give the final protein concentration of 5 mg/ml. The mixture was incubated on ice for 30 min and centrifuged at 17 000 g at 4°C for 20 min. The supernatant was collected and diluted to appropriate concentration for each oxidative phosphorylation complexes activity.

### Measurement of Oxidative Phosphorylation Complexes Activity

Measurements of mitochondria oxidative phosphorylation activities for Complex I, II, IV and ATP-synthase were carried out using the microplate immunocapture ELISA assay kit according to their respective manufacturer's protocols [15]. In general, plate pre-coated with appropriate immunocapture antibody was incubated with mitochondria extract at recommended concentration to allow immobilization of their respective complexes. Complexes activity was measured by addition of substrates solution mix provided by the kit and compound was added at concentration ranging from 0.01–10 µM in triplicate. Control wells treated with only 0.1% DMSO (v/v) and wells without mitochondria extract incubation were included as background reference. The kinetic of the complexes activity was recorded with SpectraMax M4 microplate spectrophotometer reader using the suggested measurement parameters.

### JC-1 Analysis of Mitochondrial Membrane Potential

The mitochondrial membrane potential was measured based on the potential–dependent accumulation of the cationic JC-1 dye which results in a shift of fluorescence emission from green (∼525 nm) to red (∼590 nm) due to the formation of J-aggregates [16]. Therefore, mitochondrial depolarization is indicated by a decrease in the red/green fluorescence intensity ratio. Briefly, cells were collected and suspended in 1 ml warm media at approximately 1×10^6^ cell/ml. In a control tube, 1 µl of 50 mM CCCP was added and incubated at 37°C for 5 min. Then, 10 µl of 200 µM JC-1 was added into the cells and incubated at 37°C for 30 min. Cells were washed twice with warm PBS, resuspended in 500 µl of PBS and analyzed on a FACSCalibur flow cytometer using 488 nm excitation with 530/30 nm and 585/42 nm bandpass emission filters.

### *In Vivo* Antitumor Efficacy

Tumor allografts were initiated by subcutaneous injection of 5×10^5^ 4T1 mouse mammary carcinoma in 0.1 ml RPMI 1640 media into the inguinal mammary fat-pads of mice [17]. Tumor growth was monitored and treatments were initiated when tumors reached volume of approximately 200 mm^3^. To assess the tumor growth inhibition, 4T1 tumor bearing mice were randomized into groups with at least eight animals per group. Compound **5** was prepared at 0.3 mg/ml in normal saline and treatment was administered intravenously at 5 mg/kg on staging day or 3 mg/kg every other day for six treatments (q2dx6). For control group, normal saline was given. The animal weights and tumor volume were measured three times weekly for 14 d. The tumor volume was calculated using the equation: (length×width^2^)/2 and relative tumor volume (RTV) was calculated for every tumor volume at any given time (V_T_) against the tumor volume at staging day (V_0_) using the equation: V_T_/V_0_. The RTV – time profile for each group was plotted and tumor growth delay in attaining a specified number of doublings compared to control was determined [18], [19]. Results were expressed as median ±95% confidence interval (n = 8).

### Statistical Analysis

Statistical significance were performed using one-way ANOVA *post hoc* with Bonferroni test (SPSS 16.0, IBM Corporation, Armonk, NY) and difference were considered significant when *P*<0.05.

## Results and Discussion

### Rosamine Analogs

Our previous study indicated structures *meso*-substituted with 4-iodobenzene **2** and thiofuran **5** were 5-fold more active than the phenyl substituted compound **1**. Compounds **3**, **4** and **6** were synthesized to test if *meso*-replacement with 4-bromobenzene **3**, 4-chlorobenzene **4** or 2-furyl **6** groups would affect the anticancer activity. In addition, the 4-iodobenzene **7** or 2-thiofuran *meso* substituents **8** *and* unsymmetrical piperidine/morphine amine substituents were synthesized and investigated. Rosamines **1**–**4** and **7** were not appreciably water-soluble so they were reconstituted in DMSO at 10 mM as treatment stock. Rosamines **5**, **6** and **8** were readily solubilized in aqueous media at similar concentrations, so were used without DMSO.

### *In Vitro* Antitumor Efficacy of Rosamine and NCI-60 Screen

The *in vitro* antitumor activity of rosamines was assessed using a 48 h endpoint cell viability methylthiazolyldiphenyl-tetrazolium bromide (MTT) assay against a panel of solid human tumor cell lines including breast carcinoma (MCF-7), colon carcinoma (HCT-116), oral squamous cell carcinoma (HSC-2) and nasopharyngeal carcinoma (HK-1). The IC_50_ values for these rosamines across the panel of cancer cell lines tested ranged from 0.07–1.2 µM as summarized in Table 1. From this study, rosamines bearing phenyl halide or heterocyclic moieties were significantly (*P*<0.05) more potent than phenyl substituted rosamine **1**. Halide substitution resulted in improvement of cytotoxicity; this was expected because substitution of *H* by halide is commonly used to increase compound lipophilicity that improves lipid-bilayer membrane permeability [20]. Within the halide series, cytotoxicities increase in the following order **4**>**3**>**2** (Cl>Br>I), although not statistically significant (*P*>0.05). The compounds with *meso*-heterocyclic substituents **5** and **6** were more potent than the aromatic halides **2**–**4**. Of the two compounds with *meso*-heterocycles the thiofuran-substituted **5** was slightly more active than **6**, the furan-substituted one.

**Table 1:** Antiproliferative activities of rosamine analogs against a panel of cancer cell lines.

Rosamine	Activity IC_50_ (µM)^a^				
	MCF-7	HCT-116	HSC-2	HK-1	Mean^b^
**1**	1.2±0.7	0.60±0.41	0.09±0.00	0.61±0.07	0.63 ^†^
**2**	0.59±0.12	0.39±0.07	0.35±0.05	0.29±0.03	0.41 ^‡^
**3**	0.29±0.04	0.30±0.08	0.19±0.11	0.34±0.00	0.28 ^‡,§^
**4**	0.15±0.04	0.22±0.14	0.09±0.01	0.25±0.01	0.18 ^‡,§^
**5**	0.18±0.02	0.07±0.02	0.09±0.01	0.10±0.00	0.11 ^§^
**6**	0.31±0.14	0.10±0.03	0.09±0.00	0.31±0.08	0.20 ^‡,§^
**7**	0.26±0.07	0.24±0.07	0.22±0.10	0.44±0.01	0.29 ^‡,§^
**8**	0.17±0.10	0.07±0.02	0.22±0.11	0.53±0.00	0.25 ^‡,§^

a IC_50_, the concentration of compound, which inhibits the viability by 50% as compared with control untreated cells. Values represent the mean ± SD of at least three determination assessed 48 h post-treatment using methylthiazolyldiphenyl-tetrazolium bromide assay.

b Different symbols (†, ‡, and §) indicate statistically significant differences (P<0.05) among the mean values.

Compounds **7** and **8** have a more polar combination of amine substituents than the symmetrical *bis*piperidine **2**, and they proved to be more cytotoxic (Table 1). This is consistent with our previous data for 2-methylbenzenes unsymmetrically substituted with piperidine and morpholine which showed nearly 2-fold lower IC_50_ values compared with the symmetrical hydrophobic structure containing only piperidine [10]. Meanwhile for the *meso*-thiofuran **5**, substitution of one of the piperidine groups with morpholine gives **8**, which has a slightly *decreased* activity, contrary to our expectations.

Rosamines can exhibit phototoxicity due to generation of reactive oxygen species in the presence of light. To test for this, a duplicate plate was irradiated with 5.3 J/cm^2^ of broad spectrum light for 2 h after compound treatment in parallel with a plate maintained in the dark. There were no significant differences in IC_50_ values obtained between both irradiated and non-irradiated experiments (data not shown) indicating phototoxicity was not an issue.

**Figure 3:**
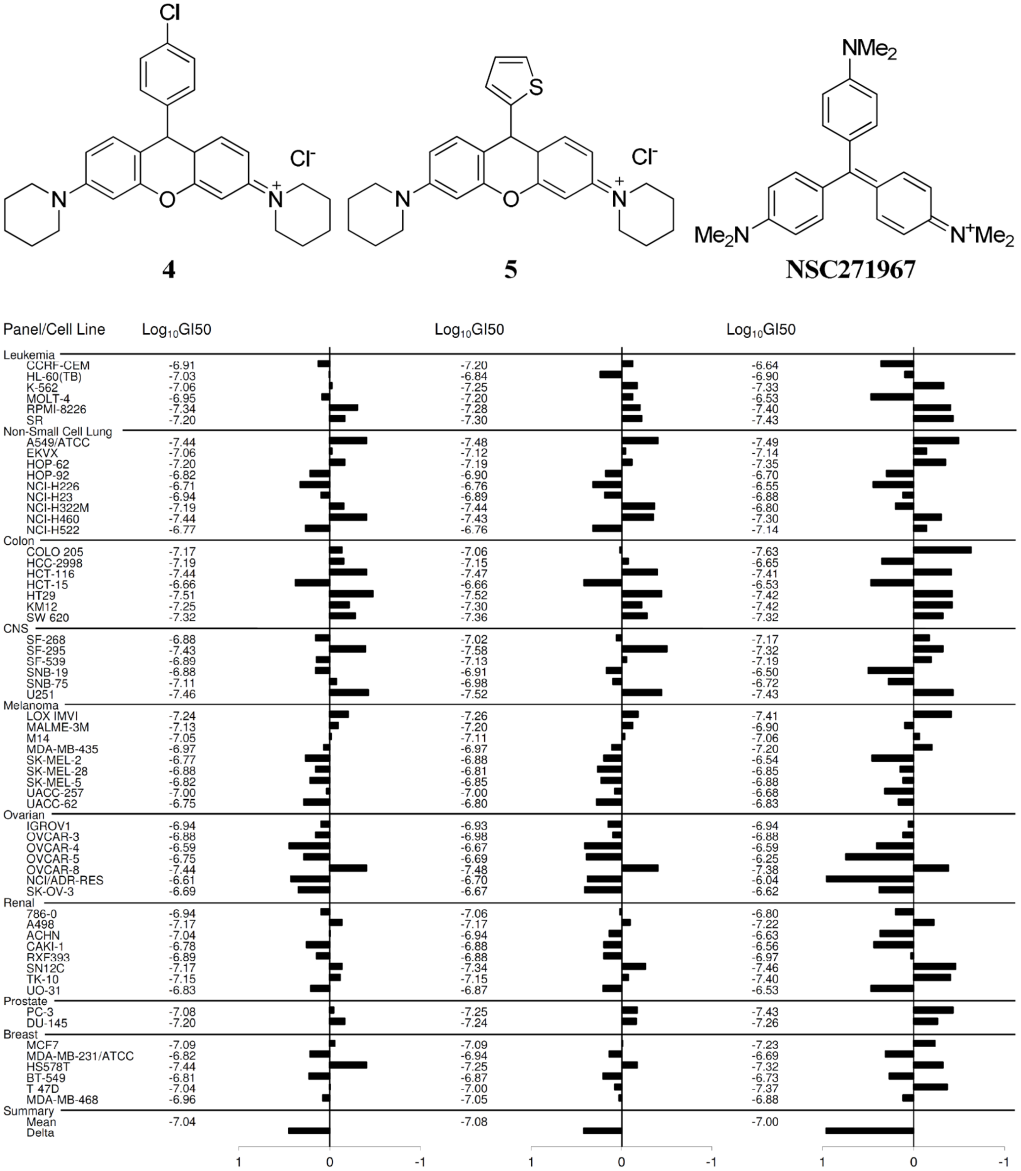
NCI-60 cell lines screen. GI_50_ (50% growth inhibition) mean graphs showing the activity patterns of **4**, **5** and methyl violet (NSC271967) in the NCI-60 cell line screens. Both the rosamines exhibited potent antiproliferative effects with Log_10_GI_50_ = −7 (GI_50_ = 0.1 µM) and were particularly more effective against colorectal cancer panel. COMPARE analyses indicated **4** and **5** have similar pattern of activity as methyl violet with Pearson correlation coefficient values of 0.767 and 0.72, respectively.

On the basis of the findings above, rosamines **4** (NSC751819) and **5** (NSC751817) were submitted for NCI-60 human tumor cell lines screen to provide more information on the growth inhibitory profiles against 60 different human cancer cell lines, representing leukemia, melanoma and cancers of the lung, colon, central nervous system, ovary, breast, prostate, and renal subpanel. This platform allows the mode of action to be inferred by comparing with the drug-activity patterns of standard agents with known targeting characteristics using COMPARE (computerized pattern-recognition algorithm) analyses [21]. The GI_50_ values generated from the NCI-60 cell lines screen indicated that both rosamine **4** and **5** exhibited potent antiproliferative effects with Log_10_GI_50_ = −7 (GI_50_ = 0.1 µM) and were particularly more effective against a sub-panel of colorectal cancer cell lines (Fig. 3). COMPARE analyses indicated that both these rosamines had similar patterns of activity with methyl violet (NSC271967), a cationic triarylmethane dye which has been formerly used in medicine for its antimicrobial, antifungal and antihelmintic properties. This class of dyes had been shown to promote mitochondrial respiratory inhibition by inhibiting ATP synthesis, dissipating mitochondrial membrane potential and inducing mitochondrial permeability transition [22], [23]. Thus the NCI-60 cell lines screen indicated the compounds tested had a signature unique to energy metabolism-targeting anticancer agents.

### Rosamines Interfered the Energy Redox

**Figure 4:**
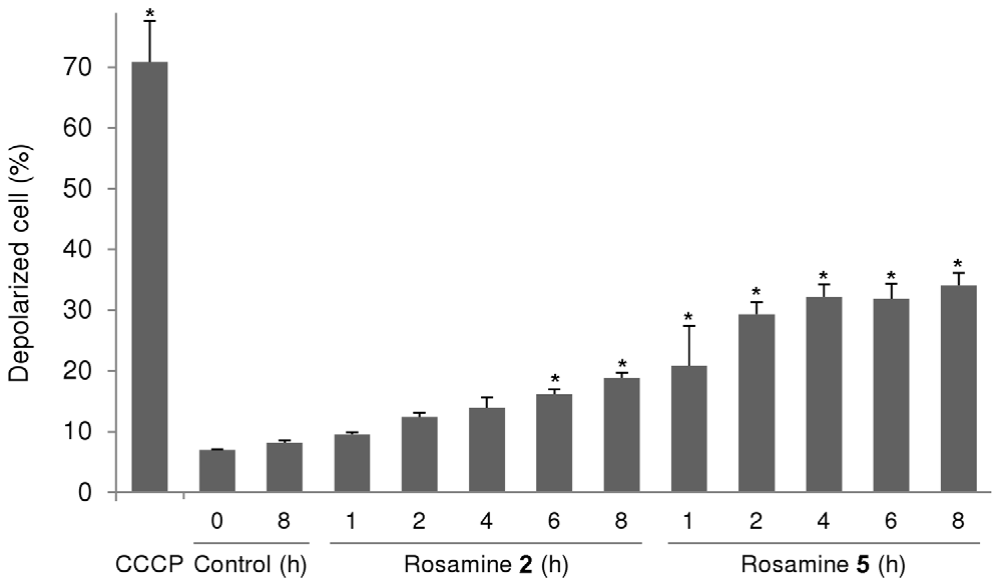
Loss of mitochondrial transmembrane potential. Representative event of mitochondrial transmembrane potential loss in HSC-2 cells treated with **2** and **5** at 0.1 µM. Following 8 h of treatment with **2** and **5**, the percentage of cell population with mitochondrial transmembrane potential loss increased to 19% and 34%, respectively. The percentage depolarized cell of untreated control at 8 h remains at 8%, while for positive control, cells treated with 5 µM of carbonyl cyanide 3-chlorophenylhydrazone (CCCP) for 5 min results in 70% depolarized cell population. *Difference with *P*-value<0.05 compared to control at 0 h.

We have previously demonstrated that rosamines primarily localize in the mitochondria [10] and that accumulation of cytotoxic DLC is known to alter mitochondrial transmembrane potentials [24], [25]. Thus, alterations of mitochondrial membrane potential caused by rosamines **2** and **5** were monitored based on the accumulation of potential-dependent JC-1 dye which resulted in a shift of fluorescence emission from green (∼525 nm) to red (∼590 nm) due to the formation of J-aggregates. From the study, HSC-2 cells treated with **2** at 0.1 µM, 9% of the cell population exhibited the onset of mitochondrial transmembrane potential loss within 1 h after treatment and gradually increased to 19% (*P*<0.05) in 8 h (Fig. 4). Meanwhile, the mitochondrial transmembrane potential loss was more prominent in HSC-2 cells treated with **5** at 0.1 µM. Approximately 21% (*P*<0.05) of the cell population was affected in the first hour and the percentage increased drastically to 34% in 8 h. For the untreated control cells, the population of depolarized cells at 8 h remained at approximately 8%. Meanwhile, cells treated with 5 µM of CCCP for 5 min resulted in loss of membrane potential in 70% (*P*<0.05) of cell population. CCCP is an oxidative phosphorylation decoupling agent which acts as a positive control for the experiment.

**Figure 5:**
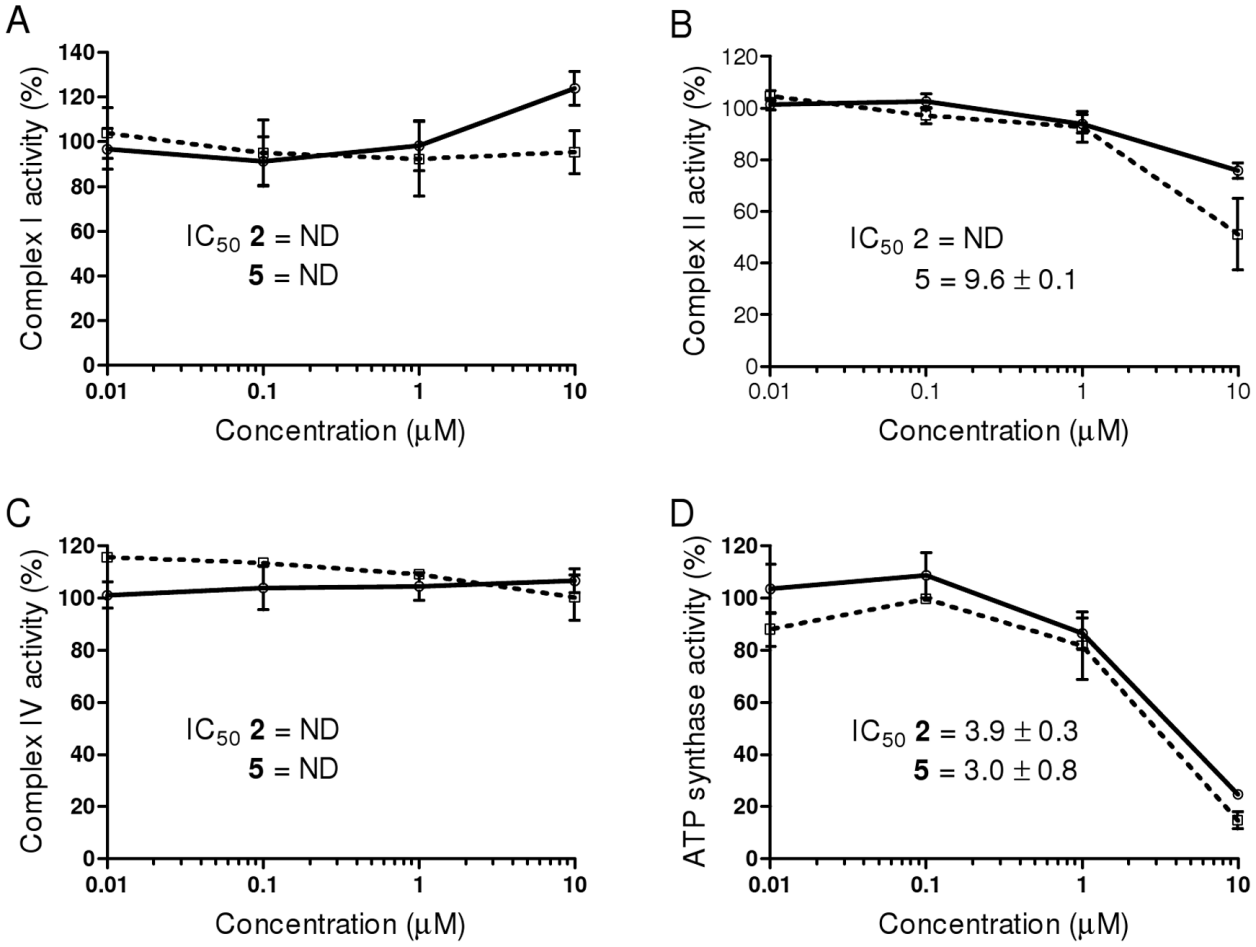
Inhibition of mitochondrial oxidative phosphorylation complexes. The dose-response inhibition of mitochondrial oxidative phosphorylation Complex 1 (A), Complex II (B), Complex IV (C) and ATP synthase (D) activities by rosamine **2** (solid line) and **5** (dotted line). The activity of Complex II was partially inhibited by **5** with IC_50_ value of 9.6±0.1 µM whereas for **2**, inhibition was observed but with undetermined IC_50_ value. Both **2** and **5** also inhibited the ATP synthase activities with IC_50_ values of 3.9±0.3 and 3.0±0.8 µM respectively. The activity of Complex I and Complex IV were not affected by the rosamines at the treated concentrations (highest at 10 µM). IC_50_ values depict concentration that inhibits the complexes activity by 50%. ND - indicate non-determined IC_50_ values based on the concentration used.

To further understand the mitochondria inhibition featured by the rosamines, their effect on the oxidative phosphorylation pathway was investigated. Using immunocapture ELISA microplate assay, the enzyme kinetics of mitochondrial redox carriers Complex I, Complex II, Complex IV and ATP-synthase were monitored upon treatment with **2** or **5**. The activity of Complex II (Fig. 5B) was partially inhibited by **5** with IC_50_ value of 9.6±0.1 µM whereas for **2**, inhibition was observed but did not reach 50% up to maximum concentrations studied. Meanwhile, both **2** and **5** displayed inhibition of ATP synthase activities (Fig. 5D) with IC_50_ values of 3.9±0.3 µM and 3.0±0.8 µM respectively. The activity of Complex I and Complex IV (Fig. 5A and 5C) were not affected by the rosamines at concentrations up to the maximum used, 10 µM. These data suggest that rosamines **2** or **5** compromise mitochondrial bioenergetics primarily by inhibiting ATP synthase, a proton-driven enzyme that produces ATP from ADP and inorganic phosphate. Similar biochemical interaction was observed in rhodamine-123 and this effect is expected as the compound has close structural similarity with the rosamines [26].

### *In Vivo* Antitumor Effect of Rosamines

**Figure 6:**
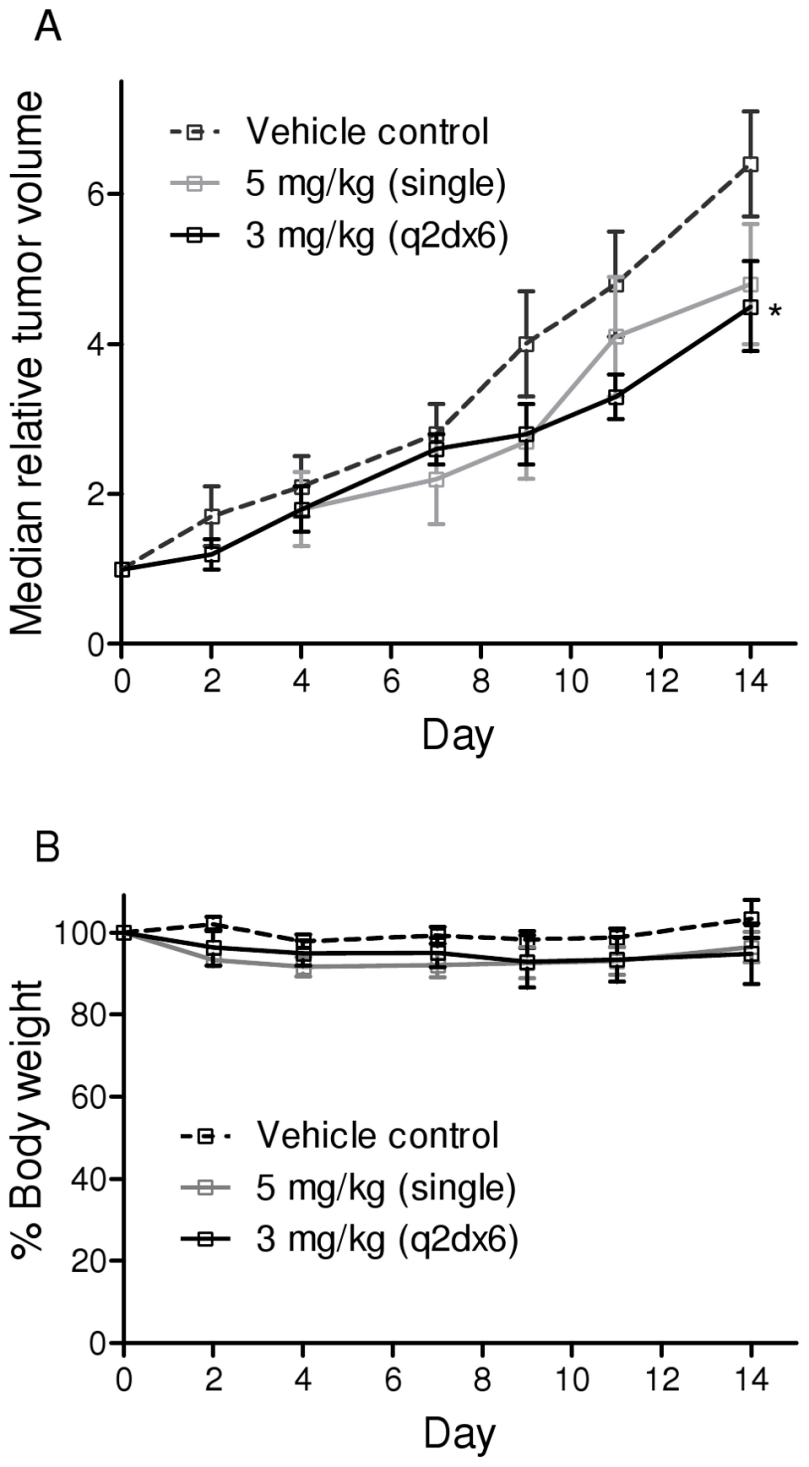
*In vivo* antitumor effects of rosamine. (A) The relative tumor volume (RTV) – time profile of 4T1 murine breast carcinoma in Balb/C mice following intravenous dosing of rosamine **5** or saline as vehicle control. Each point represents median ±95% confidence interval of RTV to staging day (n = 8). The terminal % T/C value for mice receiving 5 mg/kg and 3 mg/kg (q2d×6) of **5** were 72% and 66% respectively. The two doubling tumor growth delay (T-C)/C (dotted line, RTV = 4) for mice receiving a single bolus of 5 mg/kg and multiple doses of 3 mg/kg (q2d×6) of **5** were 22% and 38%, respectively. T and C refer to RTV for treatment and control groups, respectively. *Difference with *P*-value<0.05 compared to control animal. (B) Percentage of mean body weight of mice received 5 mg/kg or 3 mg/kg (q2d×6) of **5** compared to untreated mice. Body weight loss was observed in treatment groups but none of these mice experienced weight loss of more than 15%.

Rosamine **5** was selected for further evaluation in *in vivo* tumor model given its *in vitro* potency and ability to achieve aqueous solubility large enough for preparing an intravenous injection without the need of formulation. As illustrated in Fig. 6A, tumor growth attenuation was observed at experimental end-point (day 14) when mice received single bolus of 5 mg/kg of **5** i.v. compared control group (*P* = 0.08). The tumor growth attenuation effect is further enhanced (*P* = 0.029) when mice received rosamine treatment regimen at 3 mg/kg, once every 2 day, for 6 times (q2d×6). The median day for 4T1 tumor in attaining two-doubling growth (RTV = 4) were approximately 9, 11 and 13 days in control mice, mice receiving 5 mg/kg and 3 mg/kg (q2d×6) respectively. The determined percentage of two-doubling tumor growth delay (T-C)/C in mice treated with 5 mg/kg and 3 mg/kg (q2d×6) of **5** were 22% and 38%, respectively.

Meanwhile, the terminal percent test/control (% T/C) values calculated at the end of experiment were 72% and 66% in mice receiving 5 mg/kg and 3 mg/kg (q2d×6) respectively. The antitumor efficacy of **5** is minimal as the % T/C attained after treatment had ended is not ≤40% activity, the minimal rating for a compound to be considered active [18]. Loss of body weight (Fig. 6B) was observed in mice treated with **5** throughout the experimental period, however the reduction at any time were not statistically significant compared to the untreated mice. None of the mice suffer weight loss more that 15%, the cut-off limit set in the experiment [27].

From this study, the *in vivo* anticancer effect shown by **5** is minimal, probably because 4T1 was an aggressive tumor cell line and the dose of compound **5** used was modest. However, use of a higher concentration was not possible because unacceptable adverse effects such as weight loss, diarrhea and sudden death were observed at 10 mg/kg dose.

Even though these rosamines (i.e **2** and **5**) are more cytotoxic against cancer cells compared with normal epithelial cells (previously published data [10]), their effects on normal cells could still lead to undesirable side effects as a result of increase lactic acid accumulation in cells following decreased ATP synthase activity and a shift in metabolic pathways from aerobic to anaerobic metabolism. Accumulation of lactic acid has been shown to cause depressive cardiovascular function, cardiac arrhythmias and multiple organ failure. Therefore, risk minimization measures to deal with lactic acidosis such as those employed in the management of patients prescribed with metformin, a commonly used antidiabetic drug with similar toxic side effects, need to be outlined to prevent this serious complication [28], [29].

Another documented side effect commonly associated with DLC such as rosamines is selective accumulation of these compounds in cardiac muscle cells that can lead to fatal deterioration in the function of the heart muscle [30]. Studies have shown that cardiac muscle cells are similar to cancer cells in that both also exhibit high negative plasma membrane potentials which encourage increased uptake of DLC. In connection to this, doxorubicin which is a positively charged antracycline antitumor agent, was also shown to target the cardiac muscle cells and caused serious and occasionally fatal cardiotoxicity in a significant number of treated cancer patient [31]. However, following liposomal encapsulation of doxorubicin, cardiotoxicity was diminished in cancer patients receiving the formulation with no loss of efficacy [32]. It therefore remains to be investigated whether a similar encapsulation approach with the rosamines could also decrease their toxicity while maintaining the efficacy.

## Conclusions

The *in vitro* results demonstrated that the rosamines investigated here inhibit the proliferation of cancer cell-lines with IC_50_ value at nanomolar concentrations, with rosamine **5** being the most active. NCI-60 cell lines screen showed that these compounds are particularly effective against the colorectal cancer panel of cells and COMPARE analysis revealed a strong correlation in growth inhibitory pattern exhibited by mitochondrial respiratory inhibitor i.e. crystal violet. The anticancer effects of these compounds may have been due to their ability to compromise mitochondrial membrane potential and to inhibit the oxidative phosphorylation complexes primarily the ATP synthase which were both experimentally observed in this study. Although the preliminary *in vivo* antitumor activity of **5** is moderate against 4T1 murine mammary tumor, it may be worthwhile to test the compound in colon cancer xenograft models, for example, models based on HCT-116 and HT-29 cell lines which are both more sensitive to **5** on NCI-60 cell line screen. However further study is needed to diminish the toxic effects observed for this compound.
